# The CHOLEGAS study: multicentric randomized, blinded, controlled trial of gastrectomy plus prophylactic cholecystectomy versus gastrectomy only, in adults submitted to Gastric cancer surgery with curative intent

**DOI:** 10.1186/1745-6215-10-32

**Published:** 2009-05-15

**Authors:** Marco Farsi, Marco Bernini, Lapo Bencini, Egidio Miranda, Roberto Manetti, Giovanni de Manzoni, Giuseppe Verlato, Daniele Marrelli, Corrado Pedrazzani, Francesco Roviello, Alberto Marchet, Luigi Cristadoro, Leonardo Gerard, Renato Moretti, GIRCG (Gruppo Italiano di Ricerca sul Cancro Gastrico)

**Affiliations:** 1 General and Oncologic Surgery, Careggi Hospital, Florence, Italy; 2 First Department of General Surgery, University of Verona, Italy; 3 Unit of Epidemiology and Medical Statistics, University of Verona, Italy; 4 Department of Surgery, University of Siena, Italy; 5 Department of Surgery, University of Padova, Italy; 6 General Surgery, Pieve di Coriano Hospital, Mantova, Italy; 7 General Surgery, Ospedale Carlo Poma, Mantova, Italy

## Abstract

**Background:**

The incidence of gallstones and gallbladder sludge is known to be higher in patients after gastrectomy than in general population. This higher incidence is probably related to surgical dissection of the vagus nerve branches and the anatomical gastrointestinal reconstruction. Therefore, some surgeons perform routine concomitant cholecystectomy during standard surgery for gastric malignancies. However, not all the patients who are diagnosed to have cholelithiasis after gastric cancer surgery will develop symptoms or require additional surgical treatments and a standard laparoscopic cholecystectomy is feasible even in those patients who underwent previous gastric surgery. At the present, no randomized study has been published and the decision of gallbladder management is left to each surgeon preference.

**Design:**

The study is a randomized controlled investigation. The study will be performed in the General and Oncologic Surgery, Department of Oncology – Azienda Ospedaliero-Universitaria Careggi – Florence – Italy, a large teaching institution, with the participation of all surgeons who accept to be involved in, together with other Italian Surgical Centers, on behalf of the GIRCG (Italian Research Group for Gastric Cancer).

The patients will be randomized into two groups: in the first group the patient will be submitted to prophylactic cholecystectomy during standard surgery for curable gastric cancer (subtotal or total gastrectomy), while in the second group he/she will be submitted to standard gastric surgery only.

**Trial Registration:**

ClinicalTrials.gov ID. NCT00757640

## Introduction

The incidence of gallstones and gallbladder sludge is known to be higher in patients after gastrectomy than in general population (15–25% versus 5%, 5 years after gastric surgery) [1-4]. This higher incidence is probably related to surgical dissection of the vagus nerve branches and the anatomical gastrointestinal reconstruction [5-7].

Moreover, the risk of developing gallstones may depend on the extent of gastrectomy (subtotal or total) and lymphadenectomy (D1 or D2) [4,8-10]. Concomitant cholecystectomy is not time consuming and substantially without additional risks for the patients, because complications during another abdominal procedure are known to be minimal [11-13]. In addition theoretic advantages will be represented by the avoidance of reintervention for cholelithiasis only.

However, not all the patients who are diagnosed to have cholelithiasis after gastric cancer surgery will develop symptoms or require additional surgical treatments [2-4] and a standard laparoscopic cholecystectomy is feasible even in those patients who underwent previous gastric surgery[14].

Due to the lack of specific data and to presence of contrasting studies, some Authors[4,8] recommend to perform a prophylactic cholecystectomy at the time of gastric surgery to avoid complications and impairment of quality of life in surviving patients, while others do not[1]. At the present, no randomized study has been published and the decision of gallbladder management is left to each surgeon preference.

## Methods

### Ethical Approval

The Cholegas Study was approved by the ethical Committee of the Careggi Hospital, Florence, Italy (and from all the local Committes of the participating centers).

### Design

The study is a controlled randomized trial. The study will be performed in the General and Oncologic Surgery, Department of Oncology – Azienda Ospedaliero-Universitaria Careggi – Florence – Italy, a large teaching institution, with the participation of all surgeons who accept to be involved in, together with other Italian Surgical Centers, on behalf of the GIRCG (Italian Research Group for Gastric Cancer).

The patients will be randomized into two groups: in the first group the patient will be submitted to prophylactic cholecystectomy during standard surgery for curable gastric cancer (subtotal or total gastrectomy), while in the second group he/she will be submitted to standard gastric surgery only.

### Randomization

The randomization will be obtained through a computer-generated program (GraphPad Software, Inc, La Jolla, California, USA). The randomization, will be done before the beginning of the study and it will consist with an ordered series of numbers with a corresponding letter: A for prophylactic cholecystectomy and B for gastrectomy only. This series of numbers will be kept in the Department of General and Oncologic Surgery in Florence and after gastric cancer diagnosis if the patient fulfills the inclusion criteria, either from Florence or from any other Center throughout Italy, and signs the consent a number, progressive as in order of inclusion, will be assigned to him or her. The letter corresponding to the number, and hence the type of procedure to perform, will be notify to the operating surgeon, who will proceed accordingly.

### Statistics

#### Power Calculations

To compute sample size, the proportion of patients free from cholelithiasis was assumed to be 100% 5 years after gastrectomy plus prophylactic cholecystectomy. It is known from the current literature that the cumulative incidence of cholelitiasis 5 years after gastrectomy is around 15–25% while in the general population 5 years cumulative incidence is around 5% [1-4]. The 5-yr cumulative incidence of cholelithiasis after cholecystectomy is known to be null. Hence we assumed a 5 years cumulative incidence of 20% in patients undergoing gastrectomy alone and of 0% in patients undergoing gastrectomy + cholecystectomy.

Gastrectomy with concomitant cholecystectomy was assumed to increase cholelithiasis-free survival from 80% to 100%. Assuming a constant accrual rate and an accrual time of 2 years, a minimum follow-up period of 3 years, an overall survival probability of 50% at 5 years, 122 patients (61 in the control group and 61 in the cholecystectomy group) are necessary to ensure an 80% power with an alpha of 5%, when using a two-sided log-rank test.

Assuming a 50% drop-out rate during a 3 years follow-up allows to account for both cancer mortality and follow-up loss. These assumptions were based on the fact that competing events (deaths) will be substantial while patients lost from follow-up will be few. In a GIRCG series of 1032 patients undergoing gastrectomy plus lymphadenectomy 3 years disease-related survival was 59% (95% CI 41–60%), while no patient was lost to follow-up in the first 3 years after gastrectomy[15]. Therefore, to be conservative, we assumed a 50% loss during a follow-up period of three years, to account for both cancer mortality and loss to follow-up.

The statistical power sample calculation was carried out using PASS 2005 (Power Analysis and Sample Size, within the statistical package "NCSS 2004 and PASS 2005")[16] (see additional file 1).

#### Tests

To evaluate significance of differences between the two groups, chi-squared and Fisher's exact test will be used as appropriated for categorical variables, and the non-parametric Mann-Whitney U-test for continuous variables. To evaluate differences in the incidence of cholelithiasis, Kaplan-Meier survival curves will be computed considering as endpoint the onset of cholelithiasis itself. Survival curves will be compared by log-rank test and multivariable analysis will be accomplished by the Cox regression model. Significance level will be set at 5%. The statistical analysis will be carried out using the Statistical Package for the Social Sciences (SPSS version 13; SPSS Inc. Chicago, Illinois, USA).

### Inclusion and exclusion criteria

Inclusion criteria

• Patients suitable for curative surgery ≤ 80 years old

• Histhopatological confirmed gastric cancer (adenocarcinoma)

• Informed consent

Exclusion criteria

• Informed consent refusal

• Previous cholecystectomy

• Demonstrated cholelithiasis or biliary sludge

• Metastatic disease

• Metabolic diseases that may favour gallstones formation (hemolytic anemia, genetic hypercholesterolemia)

### Intervention

Preoperative data collection will include patient demographics and comorbidities (genitourinary, cardiac, pulmonary, gastrointestinal, renal, or rheumatologic).

The standard surgery for gastric cancer includes both total and subtotal gastrectomy with lymphadenectomy (D1, D2, D3), either laparoscopic or laparotomic. All types of reconstructions are allowed, including either mechanic or hand-sewn anastomosis. The standard open or laparoscopic cholecystectomy includes Calot's dissection with the cystic duct and artery ligated prior removal. The fundus-first technique is also allowed. Drainage tubes are left on routine basis, and withdrawn according to surgeon's preference.

### Data Collection

Patients' data files are generated containing demographic data and preoperative, operative, and postoperative information.

Pre-operative notes concern the history of gastric symptoms, the presence of associated diseases (cardiac, hypertension, diabetes, malignancy), duration of complaints (as an indication for the onset of the disease), and laboratory results of WBC count, serum bilirubin, gamma GT, and alkaline phosphatase. Ultrasound preoperative exclusion of gallbladder diseases is mandatory.

Operative data of concern are confirmation of a normal gallbladder, type of gastrectomy (subtotal or total), extent of lymphadenectomy (D1, D2 and 12^th ^node station, D3) and reconstruction. Postoperative notes of interest include the use of Total Parenteral Nutrition (TPN) or Enteral Nutrition (EN), complications, and length of hospital stay. Laboratory tests of WBC count, serum bilirubin, gamma GT, and alkaline phosphatase are also collected in postoperative days 3^rd ^and 7^th^.

Complications are classified as surgical infections (wound infection, subphrenic or subhepatic abscesses); noninfectious surgical problems (e.g. anastomotic leakage, bile duct injury, hemorrhage); remote infections (urinary or respiratory); and miscellaneous problems (e.g. pulmonary complications, deep venous thrombosis, cardiac complications, etc). The collected information are entered into a database as either continuous or categorical variables for statistical analysis.

A second surgeon (different from the one who participating in the intervention), aware of the operative findings but not the management of the gallbladder, will then assume the care of the patient. Postoperative care and ability to be discharged from the hospital will be determined by the second surgical team. This second surgical team will be blinded to the treatment of the gallbladder. The primary operative team will be in every moment available for emergent consultation.

Patient discharge will be based on good medical practice criteria: 1) apyrexia 2) absence of diseases requiring hospitalisation 3) light meals tolerance 4) return of bowel function 5) patient's compliance. A routine ultrasonography of hepathobiliary region will be scheduled after 4 and every 6 months for the first two years and yearly for the rest of the follow-up (at least 5 years) to assess possible cholelithiasis development. Routine blood check with specific hepatic tests will be collected with the same schedule. Routine oncologic follow-up in an outpatient setting are also added according to current protocols.

### Informed consent

In the informed consent patients will receive all the information about the study protocol, the confidential nature of personal data and will fill up a questionnaire before signing or refuse.

There will be not inconveniences caused to the patients. No incentives are planned for the patients regarding the operation or the follow-up. All the medical information obtained from the patients will be kept confidential among the research scientists conducting the study.

The patients will be free to withdraw from the study whenever they want without any obligation. The stopping rules will be applied also in case of newly discovered statistically significant advantages or disadvantages in one group.

### Finishing and reporting dates

The study will take approximately 1 – 3 years for the inclusion period. According to the number of gastrectomies managed monthly in all Centers, the duration of the inclusion period can be approximately 2 years to reach the number of about 122 enrolled patients.

An interim report is planned at the end of any completed follow-up period.

#### Primary aims of the study

The aim of the study is to compare the results of gastrectomy plus prophylactic cholecystectomy versus gastrectomy only in patients undergoing gastric cancer surgery with a curative intent, in terms of morbidity, mortality, operation time, hospital stay, postoperative pain, return to normal activity, quality of life, and development of biliary symptoms after 5 years from surgery.

The primary aims of our study will be:

A) To evaluate whether patients who had not a prophylactic cholecystectomy and developed cholelithiasis will eventually suffer from symptoms related to their gallstones and will need another surgical intervention 5 years after gastrectomy

B) To evaluate the incidence of cholelithiasis in patients who have undegone gastric surgery

C) To evaluate whether prophylactic cholecystectomy increases complications rate, operative time and postoperative stay.

The onset of any other complications will be recorded intraoperatively, postoperatively, at discharge, 4-months after surgery. Subsequently, visits will be scheduled every 6-months in first two years after surgery, and yearly thereafter till completion of the 5-year follow-up.

## Conclusion

Gallbladder disease is common after gastrectomy for cancer. Any improvement in this field will benefit many patients improving their quality of life and reducing morbidity, mortality, and hospital stay. Complications from cholecystectomy during another abdominal procedure are known to be minimal. The treatment group is not expected to experience dangerous complication because concomitant cholecystectomy is a very simple procedure. On the other hand, theoretic advantages will be represented by the avoidance of reintervention for cholelithiasis only.

All our patients will be informed about the study and an informed consent will be obtained. According to the best of our knowledge, there will not be any additional inconveniences caused to the patients. All the medical information obtained from the patients will be kept confidential among the research scientists conducting the study. The patients will be free to withdraw from the study, whenever they want without any obligation.

## Competing interests

None declared. Financial support by GIRCG (Gruppo Italiano di Ricerca sul Cancro Gastrico) will be provided to cover publication fee only.

## Authors' contributions

All the Authors participate to the study enrolling patients and collecting data. GV performed the statistical analysis. MF, MB, LB, EM, RM, and RM ideated and developed the study. GM, DM, CP, FR, AM, LC, and LG also contributed to the scientific accuracy of the manuscript. All the authors will be involved during data collection and interim analysis. GV performed the statistical analysis.

## Supplementary Material

Additional file 1 **ANALYSIS ASSUMING 100% of Gallstone-free survival in cholecystectomized patients**. the data provided represent the output of the PASS program for managing statistical analysis and power sample calculations. Click here for file

## References

[B1] Kobayashi T, Hisanaga M, Kanehiro H, Yamada Y, Ko S, Nakajima Y (2005). Analysis of risks factors for the development of gallstones after gastrectomy. Br J Surg.

[B2] Sanders G, Kingsnorth AN (2007). Gallstones. BMJ.

[B3] Sakorafas GH, Milingos D, Peros G (2007). Asymptomatic Cholelithiasis: Is Cholecystectomy Really Needed?. Dig Dis Sci.

[B4] Fukagawa T, Katai H, Saka M, Morita S, Sano T, Sasako M (2009). Gallstone Formation after Gastric Cancer Surgery. J Gastrointest Surg.

[B5] Rehnberg O, Haglung U (1985). Gallstone Disease Following Antrectomy and Gastroduodenostomy with or without Vagotomy. Ann Surg.

[B6] Inoue K, Fuchigami A, Higashide S, Sumi S, Kogire M, Suzuki T, Tobe T (1992). Gallbladder Sludge and Stone Formation in Relation to Contractile Function After Gastrectomy. Ann Surg.

[B7] Qvist N (2000). Review article: gall-bladder motility after intestinal surgery. Aliment Pharmacol Ther.

[B8] Wu CC, Chen CY, Wu TC, Iiu TJ, P'eng PK (1995). Cholelithiasis and cholecystitis after gastrectomy for gastric carcinoma: a comparison of lymphadenectomy of varying extent. Hepatogastroenterology.

[B9] Tomita R, Tanjoh K, Fujisaki S (2004). Total gastrectomy reconstructed by interposition of a jejunal J pou with preservation of hepatic vagus branch and lower esophageal sphincter for T2 gastric cancer without lymph node metastasis. Hepatogastroenterology.

[B10] Akatsu T, Yoshida M, Kubota T, Shimazu M, Ueda M, Otani Y (2005). Gallstone Disease after Extended (D2) Lymph Node Dissection for Gastric Cancer. W J Surg.

[B11] Watemberg S, Landau O, Avrahami R, Nudelman IL, Reiss R (1997). Incidental cholecystectomy in the over-70 age group. A 19-year retrospective, comparative study. Int Surg.

[B12] Juhasz EZ, Wolff BG, Meagher AP, Kluiber RM, Weaver AL, van Heerden JA (1994). Incidental Cholecystectomy During Colorectal Surgery. Ann Surg.

[B13] Wolff BG (1995). Current status of incidental surgery. Dis Colon Rect.

[B14] Kwon AH, Inui H, Imamura A, Kaibori M, Kamiyama Y (2001). Laparoscopic cholecystectomy and choledocholithotomy in patients with a previous gastrectomy. J Am Coll Surg.

[B15] Verlato G, Roviello F, Marchet A, Giacopuzzi S, Marrelli D, Nitti D, de Manzoni G (2009). Indexes of surgical quality in gastric cancer surgery: experience of an Italian network. Ann Surg Oncol.

[B16] Hintze J (2004). NCSS and PASS. Number Cruncher Statistical Systems.

