# Supplementary material for: The CHOLEGAS study: multicentric randomized, blinded, controlled trial of gastrectomy plus prophylactic cholecystectomy versus gastrectomy only, in adults submitted to Gastric cancer surgery with curative intent
Source: Trials. 2009 May 15;10:32. doi: 10.1186/1745-6215-10-32 (PMC2690594; doi:10.1186/1745-6215-10-32)
Supplement: Additional file 1 — ANALYSIS ASSUMING 100% of Gallstone-free survival in cholecystectomized patients. the data provided represent the output of the PASS program for managing statistical analysis and power sample calculations. [file 1745-6215-10-32-S1.doc]

**ANALYSIS ASSUMING 100% of Gallstone-free servival in cholecystectomized patients**

**Log Rank Survival Power Analysis – Advanced**

Page/Date/Time 1 23/02/2009 11.31.41

-------------------------------------------------------------------------------------------------------------------------------------------

**TWO-SIDED LOGRANK TEST**

**Numeric Results when Ha: S1<>S2**

**Surviving Surviving Accrual Follow Up Prop in**

**Power N Group 1 Group 2 Time Time Group 1 Alpha Beta**

0,80226 122 0,80000 1,00000 2,00 3,00 0,50000 0,05000 0,19774

**Summary Statements**

A two-sided log rank test with an overall sample size of 122 subjects (of which 61 are in group 1 and 61 are in group 2) achieves 80% power at a 0,05000 significance level to detect a difference of 0,20000 between 0,80000 and 1,00000--the proportions surviving in groups 1 and 2 after 5,00 time periods. Patients entered the study during an accrual period of 2,00 time periods. 50% of the enrollment was complete when 50,00% of the accrual time had past. A follow-up period of 3,00 time periods had a 50,0% loss from group 1 and a 50,0% loss from group 2.

-------------------------------------------------------------------------------------------------------------------------------------------

Base Time: 5,00

Proportion loss to follow up in group 1 during the base time: 0,50000

Proportion loss to follow up in group 2 during the base time: 0,50000

Percent of accrual time until 50% enrollment is reached: 50,00%

**References**

Lachin, John M. and Foulkes, Mary A. 1986. 'Evaluation of Sample Size and Power for Analyses of Survival with Allowance for Nonuniform Patient Entry, Losses to Follow-up, Noncompliance, and Stratification', Biometrics, Volume 42, September, pages 507-516.

**Report Definitions**

Power is the probability of rejecting a false null hypothesis. Power should be close to one.

Alpha is the probability of rejecting a true null hypothesis. It should be small.

Beta is the probability of accepting a false null hypothesis. It should be small.
